# A case with hepatic immune-related adverse events caused by nivolumab exhibiting impaired accumulation of regulatory T cells

**DOI:** 10.1007/s12328-020-01317-y

**Published:** 2021-03-05

**Authors:** Ikue Sekai, Satoru Hagiwara, Tomohiro Watanabe, Masatoshi Kudo

**Affiliations:** Department of Gastroenterology and Hepatology, Kindai University Faculty of Medicine, 377-2 Ohno-Higashi, Osaka-Sayama, Osaka 589-8511 Japan

**Keywords:** Nivolumab, Hepatic immune-related adverse effects, Regulatory T cells

## Abstract

Systemic administration of anti-programmed cell death 1 (PD-1) antibody (Ab) has achieved remarkable success in metastatic cancers. The blockade of PD-1-mediated signaling pathways sometimes cause immune-related adverse events (irAEs) due to restored anti-cancer as well as anti-self immunity. Although the liver is a preferential organ for irAEs, the immuno-pathogenesis underlying hepatic irAEs has been poorly understood. We describe a 57-year-old man with Stage IV lung cancer who underwent the first-line regimen composed of carboplatin and paclitaxel. Nivolumab treatment (3.2 mg/kg, every 3 weeks) was initiated when the disease progressed after the first chemotherapy. Sequential occurrence of irAEs involving the multiorgan systems was observed. He developed hepatic irAEs (Grade 3) after endocrine, lung, and cutaneous irAEs. Lobular hepatitis characterized by predominant infiltration of CD8^+^ T cells was seen in the liver biopsy specimens. Interestingly, defective accumulation of regulatory T cells (Tregs) expressing forkhead box protein P3 (FOXP3) was evident in this case with hepatic irAEs as compared with typical cases with autoimmune hepatitis. This case suggests that hepatic irAEs are characterized not only by lobular infiltration of CD8^+^ T cells but also by defective accumulation of FOXP3^+^ Tregs.

**Supplementary Information:**

The online version contains supplementary material available at 10.1007/s12328-020-01317-y.

## Introduction

A recent remarkable success of treating advanced cancer with immune check-point inhibitors (ICIs) highlight important roles that programmed cell death protein-1 (PD-1) and cytotoxic T lymphocyte-associated protein-4 (CTLA-4) play in the suppression of anti-cancer immune responses [1, 2]. Nivolumab and ipilimumab targeting PD-1 and CTLA-4, respectively, are the representative ICIs. These ICIs restore anti-cancer immune responses through neutralization of negative regulation mediated by PD-1 or CTLA-4-mediated signaling pathways [1, 2]. In fact, introduction of nivolumab and ipilimumab into clinical practice has improved the prognosis of advanced malignancies, especially malignant melanoma [1, 2].

Despite effective restorement of anti-cancer immunity by nivolumab and ipilimumab, the blockade of PD-1 and CTLA-4, both of which are negative regulators of adaptive and innate immunity, sometimes causes excessive immune reactions in a broad range of organs, called immune-related adverse events (irAEs) [3, 4]. Liver toxicity associated with ICIs occurs in around 5–10% of patients, [5] and thus, the liver is a preferential organ targeted by ICIs in parallel to the skin, colon, endocrine system, kidney, and lung [3, 4]. Although activation and expansion of T cells through lack of negative regulation by PD-1 and CTLA-4 is considered to underlie the immuno-pathogenesis of irAEs [3–5], molecular mechanisms accounting for the development of hepatic irAEs have been poorly understood. Regulatory T cells (Tregs) expressing forkhead box protein P3 (FOXP3) are a critical component of immune systems with pivotal roles not only in the maintenance of self-tolerance but also in the suppression of anti-cancer immunity [6]. Here, we report a case with hepatic irAEs exhibiting little accumulation of FOXP3-expressing Tregs into the liver. This case suggests possible involvement of Treg deficiency in the development of hepatic irAEs.

## Case reports

A 51-year-old man without any history of autoimmune diseases was diagnosed as lung adenocarcinoma (p-T3N0M0, Stage IIb) and received thoracoscopic right pneumonectomy in July 2012. Follow-up computed tomography (CT), which was performed in March 2017, revealed recurrence of his lung cancer in the left lobe accompanied by mediastinal lymph node metastasis. At this time point, he was diagnosed as Stage IV lung adenocarcinoma (p-T3N1M1). His surgical specimen was negative for epidermal growth factor receptor (EGFR) mutation or anaplastic lymphoma kinase 1 (ALK1) fusion oncogene. According to the guideline for Stage IV lung adenocarcinoma with intact EGFR and ALK1 [7], he was initially treated with the first-line regimen composed of carboplatin and paclitaxel from April 2017 for 4 courses followed by paclitaxel maintenance therapy from July 2017 to October 2017. These initial courses of chemotherapy were not successful and his lung cancer was judged as progressive disease (PD) based on computed tomography findings. Nivolumab was introduced from August 2018 at a dose of 3.2 mg/kg every 3 weeks.

Various kinds of irAEs occurred after the treatment with nivolumab and the severity was graded based on the clinical practice guideline following the Common Terminology Criteria for Adverse Events [version 5.0] [8].His initial irAEs involved the endocrine system as shown by the appearance of general fatigue caused by destructive thyroiditis. A significant elevation of serum free T3 (fT3, 6.2 pg/mL, normal range, 2.3–4) and fT4 (fT4, 2.1 ng/dL, normal range, 0.9–1.7) levels accompanied by a markedly reduced level of thyroid stimulating hormone (TSH, 0.02 μIU/mL, normal range, 0.5–5) was seen in September 2018. Three months later, thyroid hormone tests clearly showed hypothyroidism characterized by reduced fT3 and fT4 levels with elevated TSH levels. Further examination of his endocrine system revealed reduced serum levels of cortisol (3.3 μg/dL, normal range 6.2–19.4) and adrenocorticotropic hormone (ACTH 1.3 pg/mL, normal range 7.3–63.3), which suggested the presence of primary adrenal insufficiency despite normal levels of blood sugar, sodium and potassium. Oral supplementation of hydrocortisone (15 mg/day) and thyroid hormone (25 μg/day) were started for G1 primary hypothyroidism and G2 primary adrenal insufficiency, respectively. In addition to these endocrine toxicities, interstitial pneumonia (G1) was detected in February 2019 and then administration of nivolumab was discontinued. Moreover, skin rash judged as G3 irAE developed in March 2019. Prednisolone (PSL, 40 mg/day) with tapering schedule 5 mg/2 weeks was started after the appearance of irAEs involving the endocrine system, lung, and skin.

**Fig. 1:**
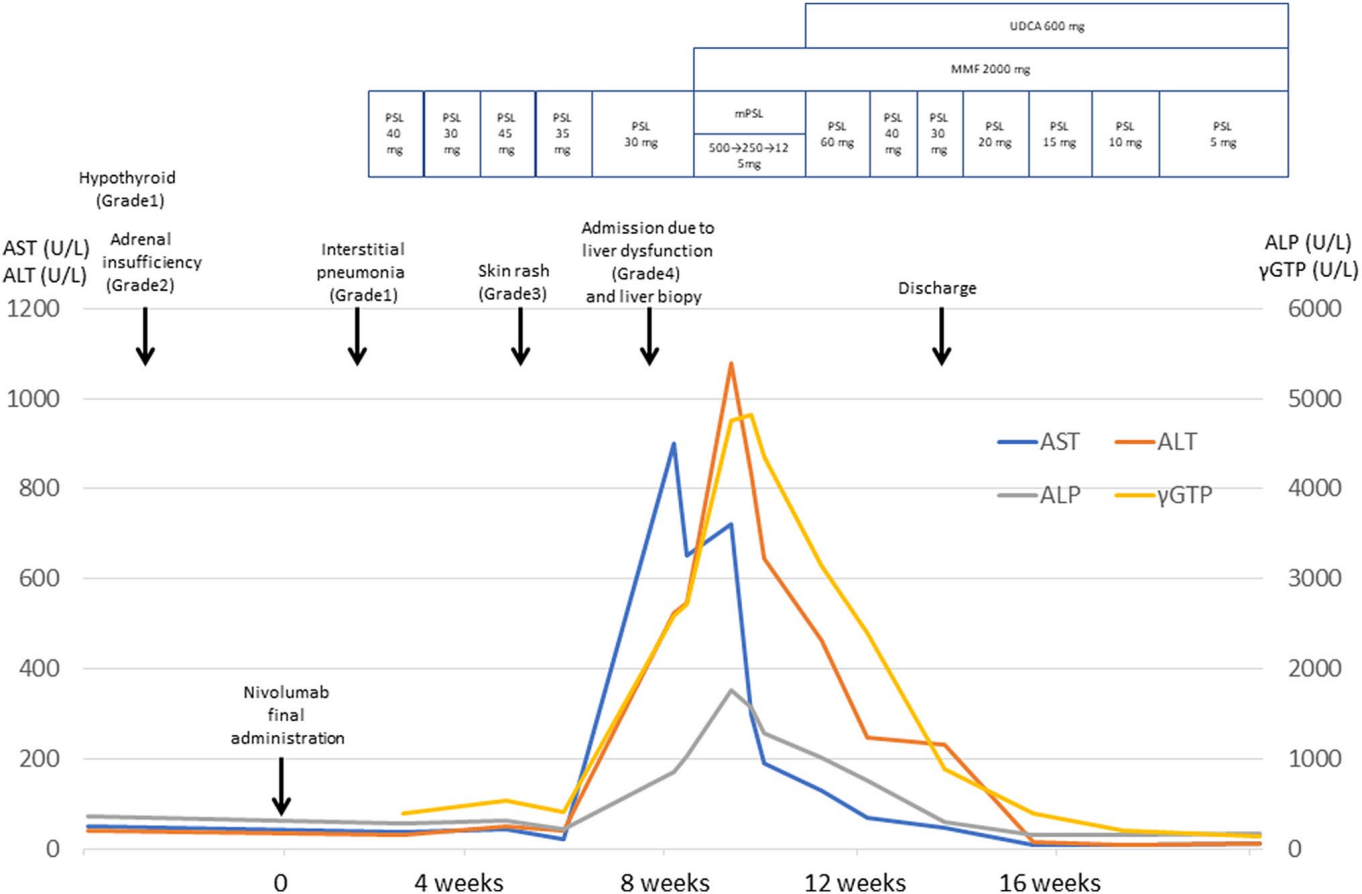
Clinical course of the patients. See the clinical presentation section of the text. *ALT* alanine aminotransferase, *ALP* alkaline phosphatase, *AST* aspartate aminotransferase, *γGTP* γ-glutamyltransferase, *MMF* mycophenolate mofetil, *mPSL* methylprednisolone, *PSL* prednisolone, *UDCA* ursodeoxycholic acid

Although improvement of skin rash was achieved after the initiation of PSL treatment, he developed liver injury as shown by a marked elevation of serum aspartate aminotransferase (AST), alanine aminotransferase (ALT), and *γ*-glutamyltransferase (*γ*GTP) (Table 1 and Fig. 1). Viral hepatitis was unlikely since viral markers for hepatitis A virus, hepatitis B virus, hepatitis C virus, cytomegalovirus, and Epstein–Barr virus were negative. Moreover, liver injury due to autoimmune hepatitis (AIH) or primary biliary cholangitis (PBC) was also unlikely as assessed by serum IgG levels and titers for anti-nuclear antibody (ANA) or anti-mitochondrial Ab. These blood examination data led us to consider the possibility of hepatic irAEs. No metastatic lesions were detected in the liver by abdominal contrast-enhanced CT. Moreover, magnetic resonance cholangiopancreatography did not reveal any abnormalities in the intrahepatic or extrahepatic bile ducts, suggesting that an immune-related cholangitis caused by ICIs was less likely [9]. To verify the diagnosis of hepatis irAEs, liver biopsy samples were subjected to hematoxylin and eosin (H&E) staining and immuno-histochemical analyses.

**Table 1:** Laboratory data on admission

Hematology		Blood chemistry			Serological tests	
WBC	6.270/μL	TP	6.2 g/dL		ANA	(–)
RBC	346 × 10^4^/μL	Alb	3.4 g/dL		AMA2	(–)
Hb	13.5 g/dL	BUN	13 mg/dL		IgG	697 mg/dL
Hct	39.0%	Cr	0.79 mg/dL		IgM	25 mg/dL
Plt	12.1 × 10^4^/μL	T-Bil	1.5 mg/dL		IgE	337 IU/mL
Neutro	85.7%	D-Bil	0.7 mg/dL		Viral marker	
Lympho	9.9%	ALP	1039 U/L		HBsAg	(–)
Eosino	0.5%	AMY	31 U/L		HBsAb	(–)
Endocrine		LDH	679 U/L		HBcAb	(–)
ACTH	1.0 pg/mL	AST	651 U/L		HCVAb	(–)
CS	6.2 μg/dL	ALT	547 U/L		HA-IgM	(–)
TSH	1.22 μIU/mL	γGTP	2718 U/L		CMV-IgM	(–)
FT4	1.1 ng/dL	CRP	10.22 mg/dL		EBVVCA-IgM	(–)
		Coagulation				
		PT		119.6%		
		INR		0.93		

*ACTH* adrenocorticotropic hormone, *CS* cortisol, *PT* prothrombin time, *INR* international normalized ratio, *AMA2* anti-mitochondrial antibody 2, *ANA* anti-nuclear antibody, *CMV* cytomegalovirus, *EBV* Epstein–Barr virus

Liver biopsy samples were subjected to H&E staining and immuno-histochemical analysis using mouse or rabbit anti-human CD3 antibody (Ab, Roche Diagnostics; Tokyo, Japan), CD4 Ab (Roche Diagnostics; Tokyo, Japan), CD8 Ab (Nichirei Bioscience; Tokyo, Japan), CD20 Ab (Roche Diagnostics; Tokyo, Japan), and FOXP3 Ab (Abcam; Cambridge, United Kingdom). Visualization of CD3^+^ T cells, CD4^+^ T cells, CD8^+^ T cells, CD20^+^ B cells, and FOXP3^+^ Tregs were performed using DakO-EnVision^+^ systems (Dako Japan, Tokyo, Japan) as previously described [10, 11]. The liver biopsy sample obtained from a typical patient with AIH (74-year-old female) was used as a disease control. Clinical parameters of this AIH patient were shown in Supplementary Table 1. As shown in Supplementary Table 1, serum levels of IgG and ANA titers were markedly elevated. Moreover, interface hepatitis accompanied by infiltration of plasma cells was evident. The AIH score proposed by international AIH group was 23 in this patient, and thus, this patient was diagnosed with definite AIH [12].

**Fig. 2:**
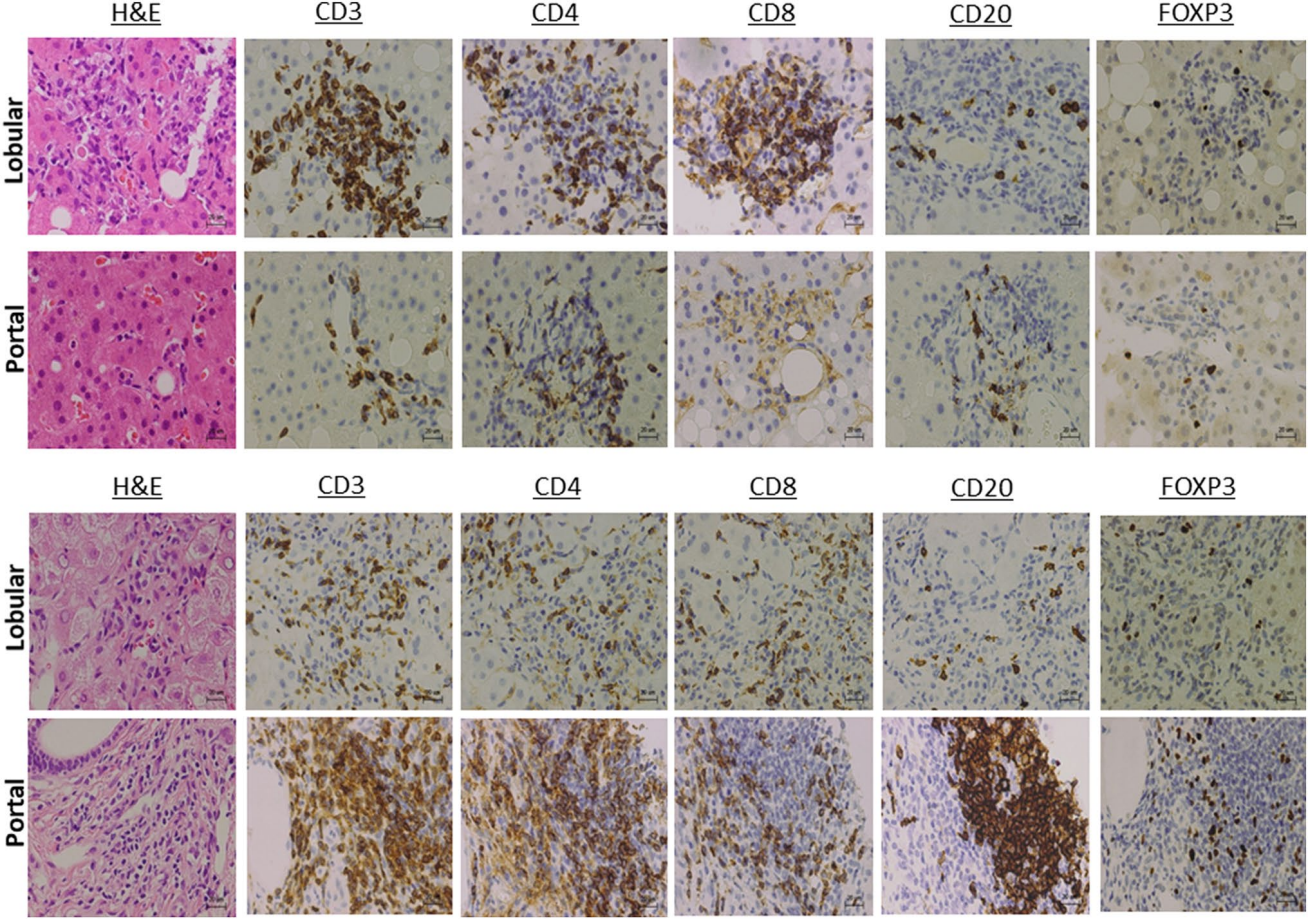
Immuno-histochemical analyses of liver biopsy specimens. Liver biopsy specimens obtained from this case with hepatic immune-related adverse effects (irAEs, 1st and 2nd line panels) and from patients with autoimmune hepatitis (AIH, 3rd and 4th line panels) were subjected to immuno-histochemical analyses using anti-CD3 Ab, anti-CD4 Ab, anti-CD8 Ab, anti-CD20 Ab, and anti-forkhead box p3 (FOXP3) Ab. Hematoxylin and eosin (H&E) staining of the portal and lobular areas (1st row). Infiltration of CD3^+^ T cells (2nd row), CD4^+^ T cells (3rd row), CD8^+^ T cells (4th row), CD20^+^ B cells (5th row), and FOXP3^+^ Treg cells (6th row) was visualized. Scale bar is 20 µm

Although lobular as well as portal inflammation was seen in the liver specimens of this patients, immune cell infiltration was predominantly seen in the liver lobes (Fig. 2, 1st and 2nd line panels). Moreover, hyperplasia of the bile duct as well as lymphocyte infiltration into the interlobular bile duct was seen in the pathological examinations, suggesting cholestasis as well as hepatocellular injury is involved in the development of hepatic irAEs in this case [13]. These pathological findings together with elevations of serum transaminases and biliary enzymes indicated that hepatic irAEs of this case was considered to be a mixed type [13].

Interface hepatitis accompanied by plasma cell infiltration was seen in the liver specimen with the AIH patient (Fig. 2, 3rd line panel). Thus, H&E staining in this case was compatible to hepatic irAEs rather than AIH since the former and latter disorders preferentially affect the liver lobes and portal tracts, respectively [5, 14].

We then performed characterization of immune cell infiltration in this case. As shown in Fig. 2, accumulation of CD3^+^ T cells was seen in the liver lobes of the patient with hepatic irAEs whereas that was seen in the portal areas of the patient with AIH. Subpopulation analysis of T cells showed predominant infiltration of CD8^+^ T cells rather than CD4^+^ T cells into the liver lobes in this case (Fig. 2, 1st line panel). AIH lesions were characterized by portal accumulation of both CD4^+^ and CD8^+^ T cells (Fig. 2, 4th line panel). Moreover, infiltration of CD20^+^ B cells was seen in the portal areas of AIH patients, but not those in this case. These immuno-histochemical analyses strongly suggest that liver injury arose from a manifestation of irAEs in that lesions are characterized by predominant accumulation of CD8^+^ T cells into the liver lobes [5]. Although Tregs expressing FOXP3 play major roles in the suppression of pro-inflammatory responses [15], their involvement in the development of hepatic irAEs has been poorly defined. Interestingly, accumulation of Tregs expressing FOXP3 was barely seen in the liver lobes of this case with hepatic irAEs whereas abundant infiltration of FOXP3^+^ Tregs was observed in the portal areas of the AIH patient (Fig. 2). Abundant infiltration of T cells expressing CD4, CD8, or FOXP3 was also seen in the other three cases with AIH in this study (data not shown). Taken together, these data suggest hepatic irAEs due to nivolumab might be characterized not only by predominant lobular infiltration of CD8^+^ T cells but also by little accumulation of FOXP3^+^ Tregs.

This patient was diagnosed as irAEs involving the endocrine system (G2), lung (G1), skin (G3), and the liver (G3). According to the guideline for hepatic irAEs [3], this patient was initially treated with intravenous administration of methyl PSL (mPSL, 500 mg/day) in combination with oral administration of mycophenolate mofetil (MMF, 200 mg/day, Fig. 1). mPSL was switched to oral administration of PSL (60 mg/day) with a tapering schedule as depicted in Fig. 1. His serum levels of transaminases became normalized 2 months after the treatment and then he was treated with oral administration of Tegafur/Gimeracil/Oteracil (120 mg/day) for advanced lung cancer.

## Discussion

Restorement of anti-cancer immunity by ICIs has dramatically improved the prognosis of patients with metastatic cancers [1, 2]. However, the blockade of PD-1 or CTLA-4-mediated signaling pathways sometimes causes irAEs, which can affect almost every organ in the body [3, 4]. In this case, administration of nivolumab led to the development of destructive thyroiditis, primary adrenal insufficiency, interstitial pneumonia, skin eruption and liver injury. Given the fact that the endocrine systems, lung, skin, and liver are preferential organs for irAEs [3–5], this case may be considered as typical irAEs due to nivolumab.

The incidence of hepatic irAEs by nivolumab alone was reported to be 6.4% [16] and liver injury by nivolumab usually develops 8–12 weeks after the initial injection [17]. Lobular infiltration of CD8^+^ T cells is one of the most characteristic findings in hepatic irAEs [5, 18, 19]. Consistent with this, lobular rather than portal hepatitis accompanied by predominant accumulation of CD8^+^ T cells was seen in this case. In contrast, AIH lesions were characterized by interface hepatitis associated with infiltration of plasma cells, CD4^+^ T cells, and CD8^+^ T cells. Thus, AIH and hepatic irAEs are distinctive disease entities in terms of the location of inflammation and effector T cell subpopulations. It should be noted, however, that molecular mechanisms accounting for such pathological differences between these two disorders have been poorly understood. In this study, we tried to elucidate the immuno-pathogenesis underlying the development of hepatic irAEs. We found that hepatic irAE lesions, but not AIH lesions were characterized by defective accumulation of FOXP3-expressing Tregs. Therefore, we assume possible involvement of defective Treg function in the development of hepatic irAEs. However, we need to be cautious regarding the interpretation of impaired Treg accumulation into the liver of hepatic irAEs. Liver biopsy samples were obtained in the middle of PSL tapering schedule in this patient. Thus, we could not completely exclude a possibility that prior PSL treatment might have affected the function of Tregs.

As mentioned above, defective accumulation of Tregs is associated with the development of hepatic irAEs in the present case. One major question arising from the present case is the molecular link between the PD-1 blockade and Treg differentiation. In this regards, neonatal thymectomy (NTx) leads to the development of much severe hepatitis in PD-1-deficient mice than in PD-1-intact mice through concurrent loss of FOXP3-expressing Tregs [20, 21]. Moreover, transient FOXP3-expressing Treg depletion in combination with systemic administration of anti-PD-1 Ab successfully induced severe hepatic irAEs in orthotopic cancer mice [22]. Thus, these previous studies together with the present case suggest the idea that PD-1 blockade may promote the development of liver injury, i.e., hepatic irAEs, in the presence of defective Treg function. Since Tregs stably express cell-surface PD-1 [15], one might assume that neutralization of PD-1-mediated signaling pathways impair Treg function. In this regards, Asano et al. provide evidence that Tregs isolated from PD-1 deficient mice display enhanced apoptosis than those from PD-1-intact mice in the presence of low doses of IL-2, a crucial cytokine for Treg proliferation [23]. Therefore, it is likely that systemic administration of anti-PD-1 Ab reduces the number of Tregs due to enhanced apoptosis. Such defective proliferation and activation of Tregs caused by nivolumab might be involved in the development of multiorgan system irAEs involving the endocrine system, lung, skin, and the liver, as seen in this case. This idea is supported by the fact that loss of function mutations in *FOXP3* causes autoimmune diseases affecting multiple organs, called IPEX syndrome [15]. Confirmation of this idea requires future studies addressing the function of FOXP3^+^ Tregs in patients with multiorgan system irAEs and solitary hepatic irAEs.

Another important question arising from this case is why lung, skin, and hepatic irAEs developed even after the withdrawal of nivolumab. In this regards, recent studies by Osa et al. provide evidence that the binding of nivolumab to cell-surface PD-1 can be detected by flow-cytometry around 6 months after the withdrawal of nivolumab [24]. Thus, such long-lasting neutralization of PD-1 may contribute to the development of irAEs even after the withdrawal of nivolumab.

The number of Tregs was greater in the liver of AIH than that of irAEs in this study. Based on this observation, we hypothesized that defective Treg function mediates hepatic irAEs. However, defective Treg function can also be involved in the development of AIH since impaired Treg function as assessed by the inhibition of effector T cell proliferation was demonstrated in AIH patients [25].

In conclusion, hepatic irAEs might be characterized not only by lobular infiltration of CD8^+^ T cells but also by defective accumulation of FOXP3^+^ Tregs. Lack of Treg-mediated immune suppression might underlie the immuno-pathogenesis of hepatic irAEs. Verification of this idea awaits further studies addressing a large number of hepatic irAEs and hepatic Treg function isolated from patients with this disorder.

## Supplementary Information

Below is the link to the electronic supplementary material.

Supplementary file1 (DOCX 17 KB)
