# Supplementary material for: A case with hepatic immune-related adverse events caused by nivolumab exhibiting impaired accumulation of regulatory T cells
Source: Clin J Gastroenterol. 2021 Mar 5;14(4):1191–6. doi: 10.1007/s12328-020-01317-y (PMC8298218; doi:10.1007/s12328-020-01317-y)
Supplement: Supplementary file 1 — Supplementary file1 (DOCX 17 KB) [file 12328_2020_1317_MOESM1_ESM.docx]

Supplementary Table. Laboratory data on admission

| Hematology | | Blood chemistry | | | Serological tests | |
| --- | --- | --- | --- | --- | --- | --- |
| WBC | 4.200/μL | TP | 8.0 g/dL | | ANA | ×320 |
| RBC | 412×10^4^/μL | Alb | 2.5 g/dL | | AMA2 | (-) |
| Hb | 14.7 g/dL | BUN | 12 mg/dL | | IgG | 4935 mg/dL |
| Hct | 42.9 % | Cr | 0.59 mg/dL | | IgM | N.E. |
| Plt | 17.3×10^4^/μL | T-Bil | 2.3 mg/dL | | IgE | N.E. |
| Neutro | 40.2 % | D-Bil | 1.6 mg/dL | | Viral marker | |
| Lympho | 43.9 % | ALP | 816 U/L | | HBsAg | (-) |
| Eosino | 1.0 % | AMY | 132 U/L | | HBsAb | (-) |
| Endocrine | | LDH | 275 U/L | | HBcAb | (-) |
| ACTH | N.E. | AST | 255 U/L | | HCVAb | (-) |
| CS | N.E. | ALT | 238 U/L | | HA-IgM | (-) |
| TSH | N.E. | γGTP | 147 U/L | | CMV-IgM | (-) |
| FT4 | N.E. | CRP | 1.009 mg/dL | | EBVVCA-IgM | (-) |
|  |  | Coagulation | | |  |  |
|  |  | PT | | 59.5 % |  |  |
|  |  | INR | | 1.23 |  |  |

ACTH, adrenocorticotropic hormone; CS, cortisol; PT, prothrombin time; INR, international normalized ratio; AMA2, anti-mitochondrial antibody 2; ANA, anti-nuclear antibody; CMV, cytomegalovirus; EBV, Epstain Barr virus; N.E., not evaluate.
